# Maternal feeding with walnuts (*Juglans regia*) improves learning and memory in their adult pups

**Published:** 2013

**Authors:** Majid Asadi-Shekaari, Abuzar Karimi, Mohammad Shabani, Vahid Sheibani, Khadije Esmaeilpour

**Affiliations:** 1 *Neuroscience Research Center, Kerman University of Medical Sciences, Kerman, I. R. Iran*; 2 *Department of Physiology, Faculty of Biology, Islamic Azad University, Branch of Arsanjan, Arsanjan, I. R. Iran*

**Keywords:** Learning, Memory, Morris Water Maze, Rat, Walnut

## Abstract

**Objective: **Walnut *(Juglans Regia)* is a domestic fruit of Iran. Walnut kernel (WK) has many beneficial constituents such as unsaturated fatty acids, antioxidants, and vitamin E. Scientific studies have shown that fatty acids and vitamin E can modulate learning and memory processes. The aim of the present work was to study effects of walnut consumption by mothers during pregnancy and lactation on learning and memory in adult rat offsprings.

**Materials and Methods:** The animals were divided into three groups: control (fed with ordinary food, 20 g daily), gestation (fed with WK, 6% of food intake during pregnancy), and gestation and lactation (fed with WK, 6% of food intake during gestation and lactation). Morris water maze test was performed for their adult offsprings.

**Results: **The results showed that there was a significant difference in learning and memory of rat offsprings between experimental and control groups.

**Conclusion: **These data may indicate that feeding mothers with WK results in improvement in learning and memory of their offsprings.

## Introduction

Walnut species are the main source of nuts in mild climate zones around the world. In Iran, *Juglans regia* L. (*Juglandaceae*) is not just an agricultural product; its fruit, leaves, stems and flowers are all used for different medicinal purposes. Walnut kernels (WK) have high concentrations of phenolic compounds, which have beneficial effects on human health because of their anticonvulsant, neuroprotective, antioxidant, and anti-atherogenic properties (Li et al., 2007 ▶; Zhang et al., 2009 ▶; Asadi-Shekaari et al., 2012 ▶; Shabani et al., 2012a). In addition, walnut kernels are a nutrient-rich food, containing plentiful of phospholipids, proteins, polyunsaturated fatty acids (PUFA), and tocopherols (Li et al., 2007 ▶). 

Different studies have shown that fatty acids and vitamin E can modulate learning and memory processes (Wu et al., 2004 ▶; Poulose et al., 2012 ▶). Walnut is extensively used in traditional medicine for treatment of various ailments such as tonifying the kidney, warming the lungs, and relaxing the bowels (Wang et al., 2004 ▶) and there are several findings that show its consumption can improve cognitive function in aged rats (Willis et al., 2009 ▶). Since some local reports are consistent with the memory enhancing effects of walnut, the aim of the present work was to study effects of walnut consumption by mothers during pregnancy and lactation on learning and memory in adult rat offspring. 

## Materials and Methods

**Animals**

Wistar adult female rats were used in this study. Kerman University of Medical Sciences Ethics Committee approved the procedures for this study (EC/KNRC/89-14). Animals were housed in standard conditions including temperature of 22±2 ºC and 25% humidity. Adult female rats, weighing 200–250 g, were housed for 2 weeks before mating at constant room temperature (22-25 °C) with light cycle of 12 h/12 h (7:00 AM to 19:00 PM) and free access to food and water ad libitum. Pairs of females were then placed with a single male rat in the late afternoon. Vaginal smears or plugs were examined the following morning. The day in which sperm was found, has been designated as the gestation day of 0 (GD 0). Then, pregnant rats were randomly divided into three groups: control (fed with ordinary food, 20 g daily) (Thomas et al., 2002 ▶), gestation (G) (fed with WK, 6% of food intake during pregnancy), and gestation and lactation (G-L) (fed with WK, 6% of food intake during gestation and lactation).

**Plant Material**

The WK was collected from Rabor area, Kerman province, Iran in September, 2010. A voucher specimen was deposited at the herbarium of Pharmacy faculty of Kerman Medical University (No. 1401-1).

**Learning and memory evaluation using Morris water maze test**

At 80 d old, spatial memory was evaluated using a modified version of the Morris water maze (MWM), in which rats learned to get away from the water onto a hidden platform (Morris, 1984). The maze consisted of an Iron circular (1.36 m in diameter and 60 cm high) filled with cloudy water with 20±1 ^o^C temperature. Animals were challenged to find a hidden platform (10 cm in diameter and 25 cm high) located 1.5 cm below water level (26.5 cm) and in the same place during the entire trial. 

The platform was always placed 30 cm beyond the rim of the pool in the center of one quadrant with respect to the distal visual cues. Swimming patterns were analyzed and the following factors were computed: latency (time spent by the rats to find the hidden platform [seconds]), time in over a quarter of, distance traveled in target quadrant (centimeters) and swimming speed (cm/s). The experiment was performed over 4 consecutive days to test their learning, when rats were located into the water facing the rim from 4 different starting points in the pool, with the platform kept in the same place and with a 1-min interval between each trial. The rats were trained in the maze during 13 trials, 4 sessions on consecutive 4 days except for the last day. The animals had to swim to find the hidden platform in 90 s (maximum time), where they were allowed to stay for 30 s. If they failed to find the platform, rats were placed on it for 10 s to associate spatial cues of the room (pictures, cabinets, and light source). One day after the last learning trial, the animals underwent a probe trial to test their long-term spatial memory. For this purpose, time spent by the rats in the target quadrant was recorded and analyzed (Shabani et al., 2012b ▶).

**Statistical Analysis**

Data were presented as mean±SEM of the mean and were analyzed using one-way analysis of variance (ANOVA) followed by the Tukey’s post hoc test and p<0.05 was considered statistically significant.

## Results

**Weight**

There was no significant difference between rat offspring’s weight in different groups.

**Effect of maternal feeding with WK on learning parameters in adult pups**

**Figure 1:**
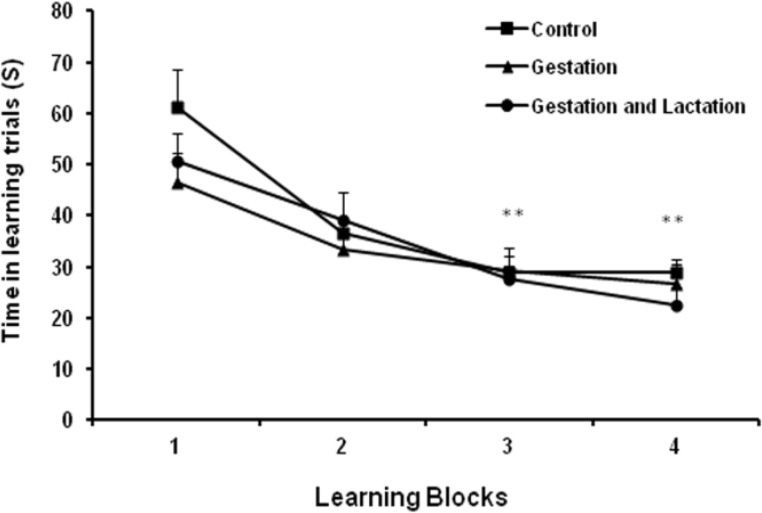
Hidden platform training in the learning phase after maternal and lactation feeding with walnut. Time spent to reach the hidden platform decreased in all groups in blocks (days) 3 and 4 compared with block 1. There was no significant difference in swimming time among all groups in block 1 and 2 in the acquisition test.^ ** ^p<0.01, as compared with block 1

**Figure 2:**
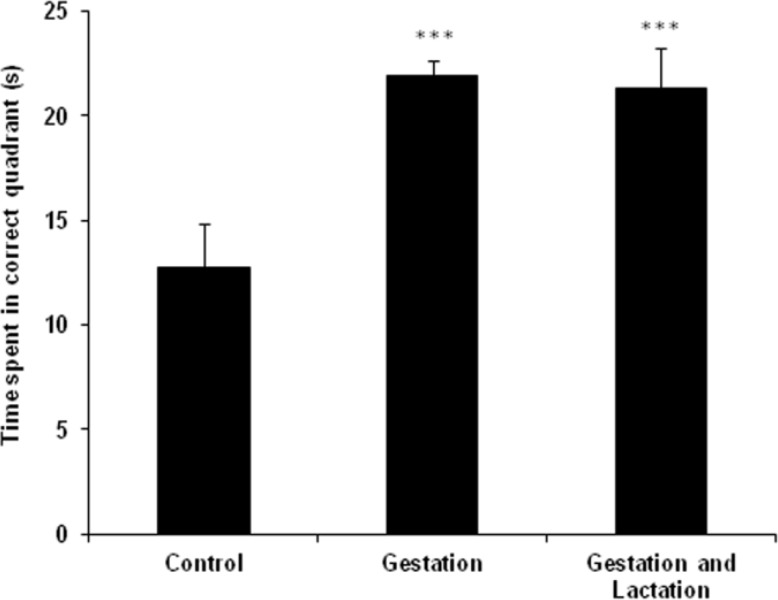
Effects of maternal feeding with WK on time in over a quarter of target of adult pups. WK adult pups presented more time (s) in over a quarter of target compared to controls (***p<0.001).

There was not a significant latency decrease for the WK (G, G-L) on the 4^th^ day compared with control (Figure 1, p>0.05). Time spent to reach the hidden platform decreased in all groups in days 3 and 4 compared with day 1 (Figure 1, p<0.01). There was no significant difference in swimming time among all groups in block 1 and 2 in the acquisition test. Although, the difference was not significant but WK adult pups demonstrated better ability in learning the position of the platform using visual cues (spatial memory). As shown in Figure 2, WK adult pups presented more time (s) in over a quarter of target compared with controls, 12.87±2.10, 21.90±0.74, and 21.26±1.95, respectively, (p<0.001).

**Figure 3:**
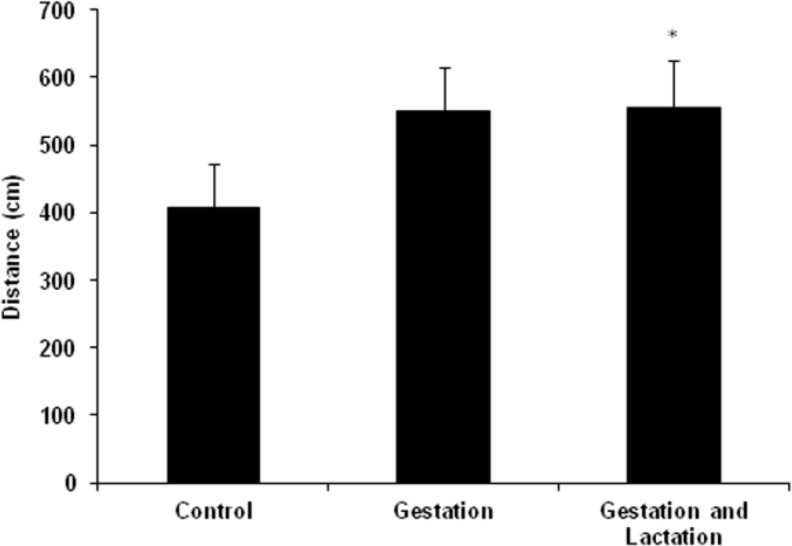
Effects of maternal feeding with WK on distance (cm) traveled by adult pups in target quadrant. *p<0.05: compared to control

**Probe trial test**

There was a significant difference (p<0.001) in time (s) spent by the rats in the target quadrant for WK adult pups (G, G-L) on the probe trial compared with control, 18.67±3.89, 25.64±3.63, and 29.04±3.95, respectively. 

## Discussion

In this study, spatial learning and memory were evaluated in adult pups of rats with WK diet or control diet throughout gestation and lactation. According to the data, animals in WK diet groups exhibited improved spatial learning and memory performance on the MWM. To our knowledge, this is the first report to demonstrate the beneficial effects of maternal feeding with WK on cognitive in adult pups of rats. 

Some studies have shown a positive effect of poly unsaturated fatty acids (PUFA) on rodent behavior (Alessandri 2004; Hooijmans, 2009 ▶). Α-linolenic acid (ALA) and linoleic acid (LA) at a 1:4 ratio improved MWM performance in young rats (Yehuda and Carasso, 1993 ▶). ALA and LA supplementation have also been shown to impact on learning and memory in mouse, resulting in improved performance on Sidman avoidance test and in light-dark discrimination learning (Umezawa et al., 1995 ▶).

Maternal fatty acid (FA) nutrition during pregnancy and lactation determines the transfer of the essential fatty acids (EFAs) (LA and ALA) and non-essential FA through the placenta and milk (Innis, 2007 ▶). Although lipid transfer through the placenta is very limited, changes in dietary FAs have implications in fetal and postnatal central nervous system (CNS) development (Herrera, 2002 ▶). In fact, the EFAs and their longer-chain, more unsaturated derivatives provide the precursors for eicosanoids (arachidonic acid, AA) and are important constituents of cell membranes, especially those in the brain tissue (docosahexanoic acid, DHA). The type and amount of FAs in the diet may alter the brain membrane lipid composition involved in cognitive behavior (Santos de Souza, 2012 ▶).

WK have high amount of PUFA which could be accounted for the effects of supplementation on cognitive performance. WK has also many beneficial constituents such as melatonin, vitamin E, folate, and polyphenols which could also contribute to the effects of WK supplementation. Melatonin has been shown to improve elevated-plus maze and passive avoidance performance in old mice (Raghavendra and Kulkarni, 2001 ▶). In addition, vitamin E and melatonin will improve MWM performance in diabetic rats (Tuzcu and Baydas, 2006 ▶). Furthermore, polyphenols in WK (its main antioxidant constituent) can improve behavioral performances (Willis et al., 2009 ▶). A large body of literatures has been suggested that dietary antioxidants can improve cognitive performance (Polidori et al., 2009 ▶; Krikorian et al., 2010; Kesse-Guyot et al., 2011 ▶). In addition, dietary antioxidants primarily influence the development of cognition and behavior during a critical period of life. Moreover, a recent study has suggested that vitamin A, vitamin E status, and vitamin E transfer rate had beneficial effects on children’s cognitive and behavior development quotients by multiple linear regression analysis (Chen et al., 2009 ▶).

According to our data, maternal feeding with WK has beneficial effects on learning and memory in adult pups of rats. These effects were more obvious when the WK supplemented to maternal diet during G-L period. Considering the large number of compounds within WK and beneficial effects of WK supplementation in the present study, the exact mechanism is not known and needs complementary works to clarify the related mechanism.
